# Lessons and Recommendations from a Pentobarbital Shortage: US and Canada 2021

**DOI:** 10.3390/ani12030365

**Published:** 2022-02-02

**Authors:** Kathleen Cooney, Lianna Titcombe

**Affiliations:** 1 Companion Animal Euthanasia Training Academy, Loveland, CO 80538, USA; lianna@caetainternational.com; 2 College of Veterinary Medicine and Biomedical Sciences, Colorado State University, Fort Collins, CO 80523, USA

**Keywords:** pentobarbital sodium, euthanasia, humane death

## Abstract

**Simple Summary:**

Pentobarbital sodium is a common animal euthanasia drug in the United States and Canada, as well as in other countries with access to it. A shortage of the drug in 2021 created a dilemma for those needing to perform the euthanasia procedure in animals facing negative welfare conditions. The reason for the shortage was attributed to chemical production issues. The veterinary community worked together to conserve what euthanasia drug was available, and to identify alternative methods that could be quickly implemented, while still adhering to safety and efficacy requirements. Through collaborative efforts, useful guidelines were created and shared with a variety of animal industries to ensure euthanasia procedures could still be facilitated when necessary. While pentobarbital sodium has increased in availability in 2022, lessons have been learned to support animal care should the shortage occur again.

**Abstract:**

In 2021, a shortage in the supply of the euthanasia drug pentobarbital sodium affected animal care professionals around the world, including in the United States and Canada. Pentobarbital sodium is the drug of choice for companion animal euthanasia in both countries. The decreased availability of pentobarbital sodium affected a number of animal care industries, forcing conservation of the drug and the use of alternative methods and other agents to facilitate humane death for all manner of animal species. Veterinary medical groups, laboratory research institutions, and the animal sheltering industry worked together to identify the best path forward to maintain routine euthanasia practices and to protect the welfare of animals. This article aims to explore the reasons behind the shortage and to highlight the necessary responses and adjustments made in order to continue providing euthanasia services in North America. Recommendations for handling future pentobarbital shortages are included.

## 1. Introduction

Pentobarbital sodium, herein referred to as pentobarbital, is the preferred drug for the euthanasia of animals in Canada and the United States of America (US) [1]. From the anesthetic drug class known as barbiturates, pentobarbital has been used for veterinary euthanasia procedures since the early 20th century. Deemed the gentlest of the “poisons” by early veterinary professionals who performed euthanasia using a number of possible agents, barbiturate overdoses lead to rapid unconsciousness, followed by the cessation of breathing and cardiac arrest [2]. Barbiturates quickly became the leading non-inhalant drug class used in animal euthanasia, as mentioned in the 1963 version of the American Veterinary Medical Association (AVMA) Euthanasia Guidelines, and in all subsequent versions [2,3]. Pentobarbital is reliable, predictable, and cost-effective. In the US, synergistic drugs have been added to pure pentobarbital to reduce the human abuse potential (e.g., phenytoin sodium, a cardiotoxic substance to speed up the cessation of electrical activity in the heart) [4].

Pentobarbital was originally only available in powdered form. End users then had to mix it precisely with the right type of diluent, and often found it unstable or at the incorrect pH for the labeled efficacy. Eventually, pentobarbital could be purchased in a ready-to-use liquid form, making it easier to work with. Other forms of barbiturates exist today; however, pentobarbital remains the most sought-after drug for animal euthanasia, in large part due to its use without the requirement for pre-euthanasia patient unconsciousness when given intravenously [3]. The chemical is produced outside of North America and is shipped to manufacturing sites in Canada and the US to create commercially-available euthanasia solutions.

In 2021, a worldwide shortage of pentobarbital began to occur. The production of both pure barbiturate products (e.g., Fatal Plus (US) and Dorminal (Canada)) and pentobarbital-combination products (e.g., Euthasol (US)) has been affected by the shortage of the chemical since late 2020. For Canada, the largest supply of the main active ingredient comes from a pharmaceutical factory in Taiwan. In December 2020, an explosion and fire occurred at the factory, killing one employee [5]. This event led to a massive disruption in the Canadian supply chain, with importers and distributors quickly taking action to preserve the remaining stock. The Canadian Animal Health Institute (CAHI) made a public service announcement in May 2021 asking Canadian veterinarians for their assistance to conserve what drug remained in stockpiles [6]. In a similar timeframe, US suppliers alerted manufacturers to chemical production and shipping delays brought about by the COVID-19 pandemic [7]. The US Food and Drug Administration (FDA) added pentobarbital to its drug shortage list in May 2021, and veterinary practices were restricted to ordering only a small amount at a time, based on purchase history [8].

The strain of the pentobarbital shortage was felt across animal-care industries, including large and small animal veterinary services, animal shelters, research facilities, academic institutions, and volunteer rescue organizations. Adjusting to new drugs and methods added complexity to an already emotional and often intricate procedure. Laboratory animal medicine and research requires that pre-determined and approved methods of euthanasia be part of study design. A change in euthanasia method is a significant research alteration that must be approved by a full committee or through designated member review [9]. Many professionals, regardless of industry, needed reassurance, support, and guidance on how to navigate the crisis.

Pentobarbital shortages have occurred in the past, but are rare. According to Vortech Pharmaceuticals, producers of the euthanasia solution Fatal Plus in Dearborn Michigan, only a few shortages have happened in the last 20 years [7]. Awareness of the shortage appeared to be noticed first by those using a large volume of the drug. This included those performing a high number of euthanasia appointments or those working with larger animals that require more pentobarbital to achieve death. Around March 2021, high volume euthanasia services/hospitals and animal shelters began to notice the declining availability of pentobarbital. The Companion Animal Euthanasia Training Academy (CAETA), an international group devoted to advanced euthanasia education, began receiving inquiries from veterinarians around the US and Canada seeking to understand the cause and expected duration of the pentobarbital shortage.

As the shortage was unfolding, governing bodies and veterinary associations avoided making formal announcements until May 2021, in an effort to reduce drug hoarding [1]. MWI Animal Health, a veterinary supply distributor owned by AmerisourceBergen, indicated a drug allocation requirement that began in May 2021, limiting the amount of pentobarbital solution that could be purchased at one time. Allocation was required by the manufacturer, as mandated by the FDA. As of December 2021, most drug distributors (those who spoke with the authors) had ended allocations and were allowing pentobarbital to be ordered without limits. To the authors’ knowledge, at no time were the US or Canada completely out of pentobarbital. Some stock, while low, always remained available. The ability to manage the shortage took creativity, collaboration, and strategy in order to navigate a serious risk to animal welfare. Sick or injured animals could suffer during the time it takes to obtain drugs, and the risk of mistakes leading to animal pain and distress increases when providers are forced to use novel techniques under less than ideal circumstances.

## 2. Veterinary Response to the Shortage

Word of the shortage spread quickly within the veterinary community’s social media platforms and through private conversations with subject matter experts and association leadership. Key opinion leaders assembled to provide information and advice. Groups like the American Veterinary Medical Association (AVMA), the Canadian Animal Health Institute (CAHI), Health Canada’s Veterinary Drugs Directorate (VDD), and recognized experts combined efforts to develop resources and guidelines on how to manage the potential crisis. The goal was to generate peer-reviewed recommendations for euthanasia that could be applied in a range of different animal care industries as fast as possible. Author communication with private veterinary practitioners revealed some were already using the alternative methods due to the shortage, and were able to share their experiences with those creating the alternative guidelines to help speed up their implementation. While research had already been conducted and published on pentobarbital alternatives for use in companion animal medicine, a trusted and authoritative body of work that could be referenced specifically in the event of a shortage had not been created before. While the AVMA 2020 Guidelines for the Euthanasia of Animals lists several alternatives, rapidly switching to a new technique without adequate training is challenging and even frightening to providers, whose primary concern is animal welfare.

With no exact substitutions available to replace what pentobarbital provided, there was a need to conserve what drug remained. Dosing and techniques were altered to use the least amount of pentobarbital possible, while still providing a comfortable and tranquil death for animals. As supplies were limited by the distributors, veterinary practices ordered what was necessary to handle immediate, short-term needs, and received whatever was available. Veterinarians and shelters were able to share supplies of the drug with other licensed professionals, as long as they followed strict drug handling and recording requirements.

Within veterinary medicine, two specialty groups appeared to be most affected by the pentobarbital shortage: emergency veterinary hospitals and end-of-life mobile veterinary companies. The concurrent COVID-19 pandemic limited the numbers of patients that could be seen by general veterinary hospitals, thus driving more patient care than usual to emergency facilities and mobile services [10]. End-of-life mobile services, which were already seeing a steady growth in patient numbers over the past decade, were hit especially hard by the increased demand for home euthanasia. In the authors’ experience and through conversations with mobile services, pet owners unwilling to leave companion pets at veterinary hospitals for euthanasia during social distancing requirements found home services a welcomed option.

Pentobarbital had to be conserved and creatively sourced. For example, some mobile veterinary end-of-life services in the US required their veterinarians to temporarily order their own drugs using individual DEA (Drug Enforcement Agency). While this is contrary to DEA recommendations, that mobile veterinary services use a single DEA number for ordering controlled substances, the practice did help meet the demand for pentobarbital beyond what traditional allocation allowed. This was not uncommon among mobile veterinary groups who were receiving unprecedented numbers of calls for home euthanasia services in both the US and Canada in 2021. Other services created multiple accounts with veterinary supply distributors and ordered novel pentobarbital products/brands they had no experience using.

Those who chose to utilize alternative drugs and methods did so with courage. It is risky to deviate from tried and trusted techniques, especially when an animal’s loved ones bear witness. Veterinarians and all who perform euthanasia have protocols they follow to prevent patient suffering and hardship for those present. Alternative drugs and methods demand adjustments, not only in the preparation and ordering of correct supplies, but also in the way that patients are readied. For example, pre-euthanasia anesthesia must be provided when using agents like potassium chloride (KCl) and T-61, a euthanasia solution containing embutramide, mebozonium iodide, and tetracaine hydrochloride [3]. Once loss of consciousness has been confirmed, the veterinarian can then proceed with euthanasia. There are also expected differences such as delayed cardiac arrest and bodily changes that are perhaps not typically seen with pentobarbital euthanasia. Depending on the alternative drug used and the administration rate, the patient may exhibit agonal breathing, muscle fasciculations, paddling, and opisthotonos stretching [11].

Because animals must be completely anesthetized when a non-barbiturate solution is given, it can be stated with confidence that any movement or other reaction is an unconscious response, and the animal remains unaware. Any witnesses must be well-informed and guided throughout the process. Choosing to end the life of a beloved companion is a difficult and heart-wrenching decision. It is essential that this final act is as smooth and peaceful as possible, and that the people present know what to expect in at least a general description (e.g., animal unconsciousness, cessation of breathing, and heart arrest).

## 3. Challenges with Animal Shelter Euthanasia

In the US, each state has its own laws governing euthanasia. Shelters had to be creative in where they ordered drugs from and perhaps switch to less-familiar products. In Michigan, the law is very specific and dictates euthanasia must be performed with a solution containing pentobarbital. Non-pentobarbital alternative options were not allowed. This meant that if a shelter had minimal to no pentobarbital, and the shelter could not quickly obtain more, they would not be able to perform euthanasia, a necessary procedure in times of unmitigable animal suffering and poor welfare. While many US and Canadian facilities reported being unaware of this issue, others were and made adjustments to the volume of pentobarbital used or worked with nearby groups to share and replenish stock, especially in high euthanasia volume locations. While pentobarbital remains the drug of choice for humane euthanasia in both countries, the availability and non-controlled status of T-61 for use in Canadian animal shelters make it a viable alternative once the requirement for pre-euthanasia unconsciousness is met.

## 4. Conserving Solution

During pentobarbital shortage, euthanasia providers had three main options, all of which could be deployed at any given time: find more solution, conserve what they had, or reach for alternative methods beyond pentobarbital (e.g., non-barbiturates or physical methods of euthanasia). Conversations within the veterinary and shelter industries revealed that most sought to locate whatever pentobarbital they could, and used it sparingly rather than try something new. This was the logical approach, especially because no one fully understood how long the shortage would last. Dr. Sharon Gwaltney-Brant, a member of the AVMA’s Panel on Euthanasia, said that in the absence of pentobarbital, “We have no 100% perfect good alternative” [12]. In the US, Vortech Pharmaceuticals posted regular updates to groups like CAETA on the arrival of the pentobarbital product into the US and when allocation restrictions were expected to be lifted. While pentobarbital remained available in small quantities in the US, Canadian veterinarians felt increased pressure to find alternatives in the face of a potential longstanding absence of the drug.

Conserving euthanasia solution was an achievable goal for many and was a reasonable way to manage the situation. It is common practice for euthanasia providers to administer an increased volume of pentobarbital to ensure death is achieved. However, this is not considered necessary as long as euthanasia is properly performed (i.e., correct intravenous placement or intraorgan access).

In the US, the standard intravenous dose of pentobarbital in animals is 85 mg/kg, a lethal dose above the LD50 of the drug, as reported by Vortech Pharmaceuticals. This equates to 1 mL per 4.5 kg of body weight as the minimum recommended dose using a 390 mg/mL solution, for all species euthanized with this drug. This is the only concentration of pentobarbital available in the US. Canadians have set the appropriate dose higher at 107 mg/kg, and as of 2021, provide differing concentrations of the solution, such as 240 mg/mL. This variation in drug concentration per ml changes how much solution is needed per patient.

While intravenous injections remain the primary route for euthanasia of companion animals, intraorgan euthanasia injections have been increasing in popularity [13]. Intracardiac, intrahepatic, and intrarenal injection techniques remove the need to access veins. While intracardiac injection requires the same dose as intravenous injection, intrahepatic (liver) and intrarenal (kidney) injection requires higher dosing to achieve death in a similar time, typically double to triple the dose [11]. The same holds true for intraperitoneal injection, which is an extremely common technique in shelter environments. During the shortage, the recommendation was to avoid techniques that required a higher pentobarbital volume, unless necessary for patient welfare and/or provider ease.

## 5. Alternative Methods

As discussions over how to handle the pentobarbital shortage progressed, subject matter experts weighed in on alternative methods. Several injectable agent substitutes were put forward, such as T-61, potassium chloride, magnesium sulfate, propofol, and intrathecal lidocaine. Physical methods such as gunshot, captive bolt, and exsanguination, were also discussed; however, to the authors’ understanding, these were only used in large animals where the methods were already familiar, and in more extreme situations with small animals when no injectable drugs were available. When pet owners were present for euthanasia, injection remained the norm. Many alternative methods require patient unconsciousness, which adds a layer of complexity for providers. When pentobarbital was the only approved method, services and facilities found it difficult to adjust. Emergency approval to allow a novel or rare technique would be necessary.

National veterinary associations dove deeply into the literature dating back to the 1950s and 1960s to find more details about the use of alternative methods. US and Canadian veterinarians connected with colleagues in other countries who had more familiarity and experience with the use of these different euthanasia methods. Countries in Europe, Africa, and South America have long been using alternatives such as T-61 and KCl due to the availability, low cost, and less-stringent reporting requirements than those required for pentobarbital. The use of high-dose propofol or other anesthetics was shared through anecdotal experience, but was enough to give some euthanasia providers the confidence needed to proceed.

While less familiar and less studied than pentobarbital, alternatives do have some advantages for longer term use rather than just during the shortage itself. Salt solutions are less costly and can be easily stored and disposed of in standard waste. Non-toxic substances proven safe for animal meat consumption are less concerning, as well as predation of any carcass remains and environmental contamination [3]. Many are non-controlled substances and have less stringent protocols for acquisition, storage, record-keeping, and disposal.

### 5.1. T-61

T-61 is a non-controlled substance approved for use in euthanasia by Health Canada’s Veterinary Drugs Directorate. It is not currently used in the US and is no longer considered to fall within the best practices of euthanasia in veterinary settings [14]. T-61 is a prescription medication composed of embutramide (a sedative), mebezonium iodide (a neuromuscular blocker), and tetracaine hydrochloride (a local anesthetic). It is administered via intravenous injection and causes death by respiratory depression and muscular paralysis, leading to cardiac arrest. It fell out of favor as a preferred drug due to the active signs of death shown by the animal and the requirement for patient unconsciousness before administration. The solution is administered only via intravenous injection at a dose of 0.3 mL per kg (0.14 mL/lb) body weight at a moderate rate [15]. The patient may exhibit muscle fasciculations, with cardiac arrest in under two minutes, at or near the same rate as a pentobarbital euthanasia [16]. T-61 is not available in many countries, including the United States.

### 5.2. Potassium Chloride (KCl)

Potassium chloride is an ionic compound salt primarily used in veterinary medicine to correct electrolyte imbalances. KCl eliminates the potassium concentration gradient in cardiac muscle, and the depolarized muscle cannot repolarize [17]. It has been a readily available and accepted agent in large animal euthanasia due to its low cost and lack of dangerous carcass residues. KCl administration requires patient unconsciousness before the injection begins. Medical grade KCl solution is more costly and requires a larger volume to cause death. The intravenous KCl dose for euthanasia is 150 mg/kg or 2 mEq/kg, although some sources list half this dose as being effective [3,13,17]. When a saturated solution containing a lethal concentration of KCl is given via vein or heart, it becomes cardiotoxic through rapid depolarization of the cardiac muscle fibers, usually in under 2–3 min. A supersaturated solution can easily be prepared by mixing the KCl salt with water in carefully measured proportions. Adding 350 g of KCl salt to 1 L of room temperature water will generate a 350 mg/mL solution. This homemade solution can be colored to avoid confusion with non-toxic substances and stored in a sealed container at room temperature. The dose for humane euthanasia using this 350 mg/mL solution is 150 mg/kg given by rapid (1 mL per second) IV bolus [17]. This equates to around 2 mls per 4.5 kg of body weight as the recommended dose using a 350 mg/mL solution.

KCl can lead to muscle fasciculations and agonal breathing in patients, which is not ideal with pet owners present. While it is a cost-effective and easy to acquire alternative, concerns over active signs of death and the risk of heart muscle recovering (lack of permanent death) have led many to avoid it. In companion animal medicine, the authors did not find this to be the preferred alternative.

### 5.3. Magnesium Sulfate (MgS04)

Magnesium sulfate is a salt that, when given intravenously in high concentrations, causes cardiac arrest by blocking peripheral neuromuscular transmission, via the reduction of acetylcholine release and inhibiting calcium influx through voltage-dependent channels, usually in under 2 min [18]. Similar to KCl, it can be purchased as a medical-grade solution or formulated into a supersaturated solution. Reported dosing is less precise, with recommendations given in a wide range at 750–1000 mg/kg through rapid intravenous bolus. Physical effects of death can include minor body stretching, muscle fasciculations, and agonal breaths. Like KCl, MgSO4 is created by dissolving 350 g of pure magnesium sulfate (Epsom salts) in 1 L of room temperature water to saturation (particulate matter accumulating in the bag/bottle) [11]. It is administered to unconscious patients until cardiac arrest is complete. A colored dye can be added to the solution to identify it for euthanasia purposes. This equates to around 10–13 mls per 4.5 kg of body weight as the recommended dose range using a 350 mg/mL solution.

Because, like pentobarbital, MgSO4 appears to work through central and peripheral nervous system suppression (although through different mechanisms), conversations with veterinary practitioners revealed more trust in its use compared to KCl. During the pentobarbital shortage, a US-based mobile end-of-life specialty service reported reaching for MgSO4 for dog and cat euthanasia daily until pentobarbital became more readily available. Their team of veterinarians used pre-mixed MgSO4 intravenously and only used pentobarbital for intraorgan injections when veins were difficult to locate in emaciated and frail patients.

When working with alternatives, veterinarians and other practitioners may feel compelled to prepare witnesses for physiologic variations compared to pentobarbital-induced death. This is reasonable; however, detailed descriptions may not be necessary for those who are not specifically concerned with the differences in drug effects and when active signs of death remain similar to what may have been observed with pentobarbital in the past. CAETA recommends gently mentioning to owners/clients that some active signs of death such as mild body stretching and deep reflexive breaths may occur, but are very normal and natural. The animal remains unaware and pain-free.

### 5.4. Anesthetic Overdoses

The AVMA provides a brief online recommendation to use lower than labeled dosing of pentobarbital sodium in combination with propofol, an anesthetic commonly used for pre-surgical anesthesia induction [19]. To the authors’ knowledge, there is little to no published data to support the irreversibility of this euthanasia method [3], yet experiential evidence is showing viability. Suggested dosing varies for propofol use. The lowest reported propofol dosing was 1.5 mg/kg in combination with pentobarbital solution at 29 mg/kg. Another protocol recommends administering propofol at 6.6 mg/kg and pentobarbital at 43 mg/kg [20]. The AVMA does not recommend reducing the pentobarbital dose by more than 50%, though individual practitioners have reported success at lower doses when used in conjunction with propofol [21]. Until further data reveal the irreversibility of these drug combinations, the authors advise caution in using lower than labeled doses of pentobarbital to avoid confusion and accidental reversible death. Other potential anesthetics used for anesthetic overdosing can be found in the AVMA 2020 euthanasia guidelines.

### 5.5. Intrathecal Lidocaine

Lidocaine (2% in solution) is a local anesthetic that, when injected into cerebrospinal fluid, acts on the medullary region of the central nervous system causing loss of consciousness, respiratory arrest, and cardiac arrest. This method has been researched in equids [22] and remains formally untested within the small animal population. Lidocaine is inexpensive and readily available in most countries. Intrathecal injections require patient unconsciousness before administration [22]. In a time of preferred euthanasia solution shortage, this alternative may prove beneficial, however the skill required to adequately perform the technique may render it less preferred.

## 6. Summary

While the pentobarbital shortage may extend into late 2022, manufacturers are currently working to restore operations and improve the supply chain. According to Health Canada, additional supplies of pentobarbital sodium have now been secured, with adequate supply expected to cover normal Canadian demand through the end of 2021 [23]. The US appears to have reestablished normal supply at the time of writing this article. Government drug directorates are seeking alternate supplies of pentobarbital that might not otherwise have been considered. Veterinary governing bodies will continue to provide updated information as the crisis unfolds. In the meantime, veterinarians, shelter personnel, research facilities, and other stakeholders around the world are leveraging alternatives as needed to ensure the timely delivery of euthanasia and improving the comprehension of such techniques to improve animal welfare. Practitioners are advised to create clinical practice guidelines to manage any drug shortage and improve response time, plus ensure veterinary personnel are trained in the use of alternative methods. In conclusion, experts have come together to share knowledge and experience from different disciplines, sectors, and countries. Navigating this crisis has led to considerable collaboration, demonstrating the solidarity of those invested in animal welfare. The veterinary community can take pride in recognizing its aptitude and resilience when faced with yet another challenge, and feel better prepared should the shortage occur again.

## Data Availability

Not applicable.
